# The flavoprotein Mcap0476 (RlmFO) catalyzes m^5^U1939 modification in *Mycoplasma capricolum* 23S rRNA

**DOI:** 10.1093/nar/gku518

**Published:** 2014-06-17

**Authors:** Carole Lartigue, Anne Lebaudy, Alain Blanchard, Basma El Yacoubi, Simon Rose, Henri Grosjean, Stephen Douthwaite

**Affiliations:** 1 INRA, UMR 1332 de Biologie du Fruit et Pathologie, F-33140 Villenave d'Ornon, France; 2 Université de Bordeaux, UMR 1332 de Biologie du Fruit et Pathologie, F-33140 Villenave d'Ornon, France; 3 Department of Microbiology and Cell Science, University of Florida, FL 32611, USA; 4 Department of Biochemistry and Molecular Biology, University of Southern Denmark, DK-5230 Odense M, Denmark; 5 Centre de Génétique Moléculaire, UPR3404, CNRS, Associée à l'Université Paris Sud 11, FRC 3115, F-91190 Gif-sur-Yvette, France

## Abstract

Efficient protein synthesis in all organisms requires the post-transcriptional methylation of specific ribosomal ribonucleic acid (rRNA) and transfer RNA (tRNA) nucleotides. The methylation reactions are almost invariably catalyzed by enzymes that use S-adenosylmethionine (AdoMet) as the methyl group donor. One noteworthy exception is seen in some bacteria, where the conserved tRNA methylation at m^5^U54 is added by the enzyme TrmFO using flavin adenine dinucleotide together with *N*^5^,*N*^10^-methylenetetrahydrofolate as the one-carbon donor. The minimalist bacterium *Mycoplasma capricolum* possesses two homologs of *trmFO*, but surprisingly lacks the m^5^U54 tRNA modification. We created single and dual deletions of the *trmFO* homologs using a novel synthetic biology approach. Subsequent analysis of the *M. capricolum* RNAs by mass spectrometry shows that the TrmFO homolog encoded by Mcap0476 specifically modifies m^5^U1939 in 23S rRNA, a conserved methylation catalyzed by AdoMet-dependent enzymes in all other characterized bacteria. The Mcap0476 methyltransferase (renamed RlmFO) represents the first folate-dependent flavoprotein seen to modify ribosomal RNA.

## INTRODUCTION

The stable ribonucleic acids (RNAs) of all organisms are post-transcriptionally modified to facilitate the array of functions they carry out during protein synthesis (1,2). Methylation at the C5-position of uridine (forming m^5^U) is an RNA modification commonly found in Bacteria and Eukaryota, as well as in some Archaea (3). Most m^5^U RNA modifications are catalyzed by methyltransferases that belong to the COG2265 enzyme cluster and use S-adenosylmethionine (AdoMet) as their methyl group donor (4,5). For instance, there are three m^5^U modifications in *Escherichia coli* tRNAs and rRNAs, and these are added by three COG2265 paralogs: RlmC and RlmD respectively modify 23S rRNA at m^5^U747 and m^5^U1939 (6,7) and TrmA modifies tRNAs at m^5^U54 (8). *Bacillus subtilis* has the same m^5^U modifications, but these are added in a distinctly different manner. In *B. subtilis*, both m^5^U747 and m^5^U1939 are catalyzed by a single AdoMet enzyme, RlmCD (9), while m^5^U54 in tRNA is added by the COG1206 flavoprotein TrmFO, which methylates using *N*^5^,*N*^10^-methylenetetrahydrofolate in conjunction with reduced flavin adenine dinucleotide, FADH_2_ (10). Database searches suggest that other bacteria possess orthologous folate-dependent methyltransferases (11) and the few characterized examples also methylate tRNAs (10,12–14). All previously characterized rRNA methyltransferases, irrespective of their nucleotide targets, are dependent on AdoMet as a cofactor (3,5).

We have investigated whether the present picture of RNA modification mechanisms is also applicable to bacteria such as mycoplasmas with minimal genomes. The stable RNAs of mycoplasmas possess many characteristic bacterial modifications, including m^5^U, and certain mycoplasmas have more than one copy of *trmFO*-like genes (15). The presence of multiple *trmFO* copies is particularly intriguing because the extensive reduction of mycoplasma genome size during evolution is generally taken as a credential for the functional importance of any remaining genes (16–18). Here we describe the development of an offshoot of a synthetic biology approach (19–21) to determine how the products of such genes might function. This approach circumvents many of the technical challenges that have hindered genetic engineering of mycoplasmas, and facilitates the previously impracticable task of creating clean multiple gene deletions in a mycoplasma chromosome.

**Figure 1.**
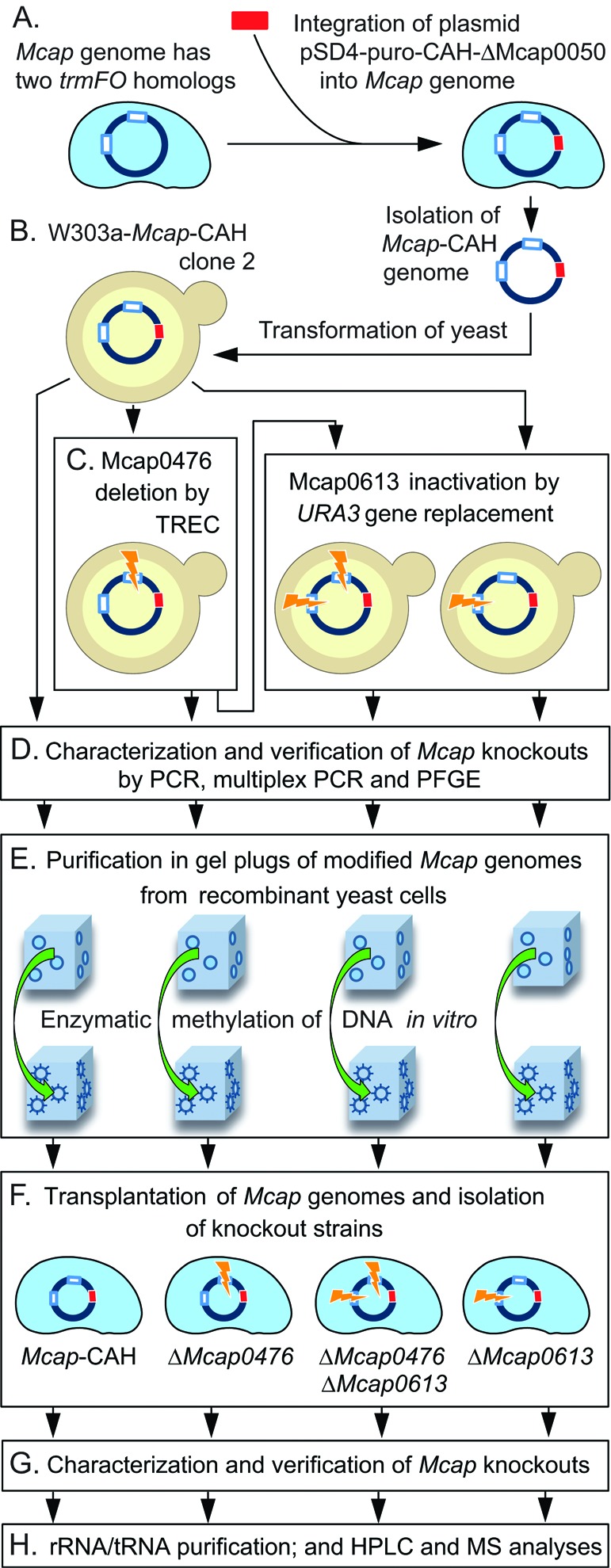
Synthetic biology strategy for manipulation of the *Mcap* genome. (**A**) The *Mcap* genome encodes two *trmFO* homologs, Mcap0476 and Mcap0613 (white rectangles). Strain *Mcap*-CAH was created by integration of the plasmid pSD4-puro-CAH*-*Δ*Mcap0050* (red rectangle) into the *Mcap* chromosome (21). Plasmid pSD4 contains the yeast centromere CEN6, an autonomously replicating sequence ARSH4, and the auxotrophic marker HIS, for propagation and selection of the *Mcap-*CAH genome in yeast, as well as a puromycin resistant marker for selection in mycoplasma. (**B**) Yeast cells were transformed with the intact *Mcap*-CAH genome. (**C**) Mcap0476 and/or Mcap0613 were excised by transforming yeast cells with PCR-generated cassettes containing fragments of the targeted genes. Mcap0476 was cleanly deleted by the TREC method (tandem repeat coupled with endonuclease cleavage, Figure 2A), an approach that can be reiterated to inactivate multiple genes in the same chromosome. Mcap0613 was removed by insertion of the *URA3* gene (Figure 2B). (**D**) Gene knockouts were verified by PCR, and the integrity of the *Mcap* chromosomes was checked by multiplex PCR and PFGE (Supplementary Figures S2 and S3). (**E**) Recombinant *Mcap* chromosomes were extracted from yeast cells in agarose gel plugs, and the DNA was methylated *in vitro* to avoid restriction upon (**F**) transplantation back into *Mcap* cells to replace the wild-type genome. (**G**) The structures of the *Mcap*-recombinant genomes were verified (Supplementary Figure S4) before (**H**) analysis of the mycoplasma tRNA and rRNA modifications by HPLC and mass spectrometry (MS).

*Mycoplasma capricolum* subsp. *capricolum* (henceforth abbreviated as *Mcap*) possesses a relatively small genome of ∼1.0 Mb that encodes two *trmFO* homologs, Mcap0476 and Mcap0613 (Supplementary Figure S1), but no homologs of *rlmCD* (15). *Mcap* tRNAs and rRNAs have been reported to be devoid of AdoMet-dependent m^5^U (22–24), and while our own (previously unpublished) analyses confirmed the lack of this tRNA modification, we surprisingly found clear evidence of m^5^U in *Mcap* rRNA. To investigate whether one of the *Mcap trmFO* homologs is responsible for rRNA methylation, the *Mcap* chromosome was equipped with genetic elements to enable its relocation and stable replication in yeast (25) and the Mcap0476 and Mcap0613 genes were deleted individually and in combination (Figure 1). The mutagenized *Mcap* chromosomes were then moved back to *Mcap* recipient cells replacing the wild-type chromosome. RNAs from the *Mcap* mutants were purified and analyzed by mass spectrometry (MS) and high performance liquid chromatography (HPLC) to locate the m^5^U in 23S rRNA and identify the methyltransferase catalyzing this modification. These findings link for the first time a folate-dependent flavoprotein with an rRNA target, and show that two distinct m^5^U modification mechanisms evolved independently within the Bacteria to methylate the same rRNA nucleotide.

## MATERIALS AND METHODS

### Bacterial and yeast strains and culture conditions

*Escherichia coli* (*Electromax DH10B* from Invitrogen) [F^−^-*mcrAΔ(mrr-hsdRMS-mcrBC), ф80dlacZ, ΔM15, ΔlacX74, recA1, endA1, araD139, Δ(ara, leu)7697, galU, galK, λ^−^, rpsL, nupG*] served as host strain for cloning experiments and plasmid propagation. Plasmid-transformed *E. coli* cells were grown at 37°C in Luria-Bertani (LB) broth or on LB agar supplemented with ampicillin at 100 μg/ml.

Wild-type *M. capricolum* subsp*. capricolum* (*Mcap*-wt) strain California Kid^T^ (ATCC 27343) and the engineered strain *Mcap*-CAH (Figure 1 and Supplementary Figure S2) were used in this study. *Mcap*-CAH (formerly *Mcap*ΔRE cl17.5) was created by integration of the plasmid pSD4-puro-CAH*-*Δ*Mcap0050* into the *Mcap*-wt chromosome (21). Plasmid integration inactivated the CCATC-restriction system in addition to providing a puromycin resistant marker for selection in mycoplasma, as well as the yeast centromere CEN6, an autonomously replicating sequence ARSH4, and the auxotrophic marker HIS, for propagation and selection of the *Mcap-*CAH genome in yeast. Integration at the *Mcap0050* locus was verified by Southern blot analysis and by loss of restriction activity (21). The *Mcap*-wt and *Mcap*-CAH strains were grown at 37°C in SP4 medium (26), supplemented with 8 μg puromycin/ml for *Mcap*-CAH. For transplantation experiments, *Mcap*-wt recipient cells were grown at 30°C in super optimal broth (SOB) supplemented with 17% (v/v) fetal bovine serum, glucose at 10 g/l, 0.002% (w/v) phenol red, and penicillin at 0.5 μg/ml (SOB (+) medium). Prior to RNA analyses, *Mcap* cells were grown in 3 l of SP4 medium and harvested in mid-log phase by centrifugation (30 min, 12 000 g, 4°C). Cells were washed three times in 8 mM HEPES and 280 mM sucrose, pH 7.4 and pellets were stored at −80°C.

*Saccharomyces cerevisiae* strain W303a (MATa *his3-11, 15 trp11, leu2-3,112 ura3-1, ade2-1, can1-100*) was grown at 30°C in YDPA medium (Clontech) (27). After transformation with polymerase chain reaction (PCR) fragments, yeast strains harboring mycoplasma genomes were grown in minimal SD Base medium (Clontech) with the supplements −HIS DO (SD minus HIS medium, Clontech), or with −HIS −URA DO (SD minus HIS minus URA medium, Clontech). For counter-selection, yeast cells were grown in SD − HIS medium supplemented with 5-fluoro-orotic acid (5-FOA) (28).

### Yeast transformation with *Mcap*-CAH genomic DNA

Whole intact *Mcap*-CAH genomic deoxyribonucleic acid (DNA) was isolated in agarose plugs using the CHEF Mammalian Genomic DNA Plug Kit (Bio-Rad) with some procedural modifications (21) and was used to transform *S. cerevisiae* W303a (29).

### *Mcap* genome manipulation in yeast

**Figure 2.**
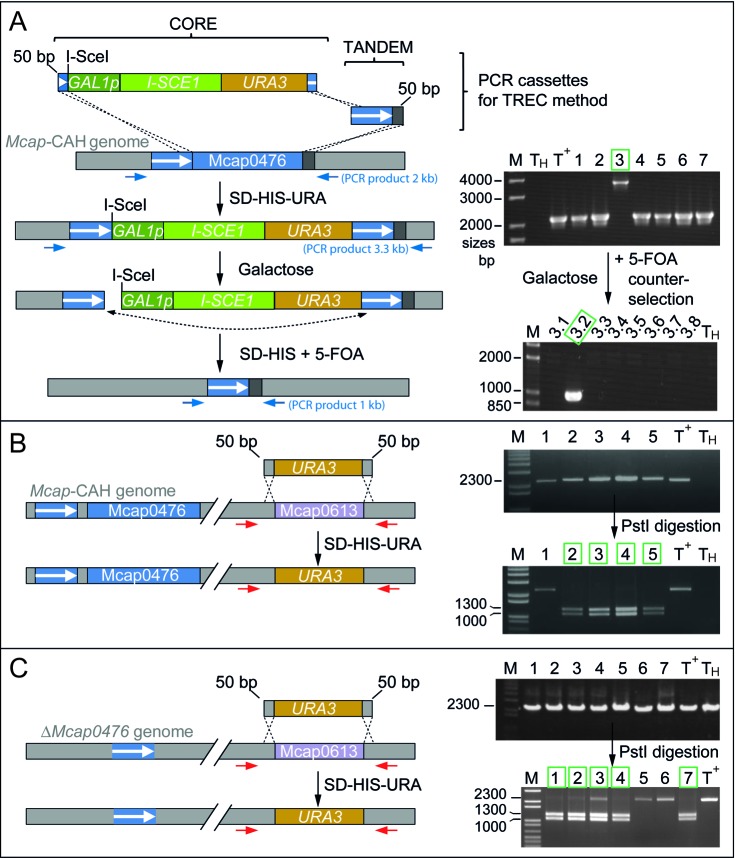
Inactivation of the Mcap0476 and/or Mcap0613 genes in *Mcap* genomes maintained in yeast. (**A**) Strategy for inactivation of Mcap0476 using the TREC method (30) whereby two overlapping cassettes (CORE and TANDEM) containing Mcap0476 sequences were introduced into yeast spheroplasts carrying the *Mcap*-CAH genome. Yeast recombinants were selected on solid SD-HIS-URA medium. Homologous recombination with three crossing-over events formed direct tandem repeats (white arrow, blue background) and resulted in the replacement of Mcap0476 by the CORE and TANDEM cassettes. Replica-plating of yeast recombinants on galactose solid medium induced expression of I-SceI followed by cleavage at the I-SceI site. This double-strand break increased the frequency of homologous recombination events (dash line) between the tandem repeats followed by cassette excision, which was selected for by plating on solid medium containing 5-fluoroorotic acid (SD−HIS + 5-FOA). Yeast recombinants were analyzed using the PCR primer pair TrmFO15a and TrmFO16 (Supplementary Table S1) positioned on either side of Mcap0476 (blue arrows). PCR products of 2 kb showed that Mcap0476 was still intact in clones 1, 2, 4, 5, 6 and 7, while the 3.3 kb band from clone 3 revealed the desired insertion of CORE and TANDEM cassettes. Subsequent excision of the cassettes is indicated by the 1 kb PCR band from subclone 3.2. This subclone, renamed W303-Δ*Mcap0476*, was analyzed further by multiplex PCR and PFGE (Supplementary Figure S3). (**B**) Mcap0613 was deleted in *Mcap-*CAH by conventional gene replacement with a *URA3* cassette. Yeast spheroplasts housing the *Mcap*-CAH genome were transformed with the cassette to promote homologous recombination involving two cross-overs and resulting in the replacement of Mcap0613 by *URA3*. PCR reactions were performed on candidate yeast clones with the primer pair TrmFO25b and TrmFO26 (red arrows) (Supplementary Table S1). The *URA3* and Mcap0613 genes both produce fragments of 2.3 kb, and were discriminated by cleavage at PstI in *URA3* into 1.0 and 1.3 kb bands. Positive W303-Δ*Mcap0613::URA3* clones (2, 3, 4 and 5) are boxed in green. (**C**) The same procedure was applied to the W303-Δ*Mcap0476* strain to produce the double knockout W303-Δ*Mcap0476/*Δ*Mcap0613*::URA3. Positive clones (1–4 and 7) are boxed in green. Positive clones were taken further for multiplex PCR and PFGE analyses (Supplementary Figure S3). M, DNA size ladder (Invitrogen); T^+^, DNA from *Mcap*-wt cells; T_H_, negative control, no DNA.

The Mcap0476 gene was seamlessly deleted in the *Mcap*-CAH genome using the TREC (tandem repeat coupled with endonuclease cleavage) method (30), which involved homologous recombination with two PCR cassettes, CORE and TANDEM (Figure 2A). The CORE cassette was amplified as a 2.5-kb PCR product using the primers 5′-ATGAGATTAATATATTTAAAGATTTAGACAGAGAGCAAAATAATGAATAATAGGGATAACAGGGTAATAC (TrmFO11) and 5′-GTATAGTCATTGTTTAAAGCTCCGGGTAATAACTGATATAATTAAATTGAAG (TrmFO12). This cassette encodes *URA3* and the I-Sce1 endonuclease. The *I-Sce1* gene is under the control of a GAL1 promoter (*GAL1p*) with an 18-bp recognition site (I-SceI) located immediately upstream. The CORE cassette is flanked on its 5′-side by the 50 bp found immediately before the start of Mcap0476, and on its 3′-side by 50 bp identical to the sequence 250–300 bp upstream of Mcap0476 (Figure 2A). The TANDEM cassette (350 bp) was PCR amplified from the *Mcap* genomic DNA using the primers TrmFO13 (5′-CTTCAATTTAATTATATCAGTTATTACCCGGAGCTTTATTCAATGACTATAC) and TrmFO14 (5′-GAGTAAAATTTATCATTACTTTG-ATATTTATTTTCTTGTTCTAAAATACCTTATTCATTATTTTGCTCTCTG). The TANDEM cassette contains the 300 bp sequence upstream of Mcap0476 plus the 3′-terminal 50 bp sequence of Mcap0476 at its 3′-end.

Inactivation of Mcap0613 was achieved using a standard gene replacement method. Briefly, the *URA3* marker (1.2 kb) was PCR-amplified from the yeast centromeric plasmid pRS316 (31) using the primers TrmFO27 (5′-TTTCTGATTATAAATAGTTAAGTTGGTAAGTATGAAAACAATAAGAATCACTACATCGATTCTATGTCTTACC) and TrmFO28 (5′-CAGATAATTCAAAACCACCAATCATATCAACTAAAGCTCAATTAATTCTCGGGTAATAACTGATATAATTAAATTG). The *URA3* cassette was amplified by PCR to give a product consisted of the *URA3* gene flanked by 50 bp sequences identical to the ends of the Mcap0613 gene (Figure 2B).

Yeast cells containing the *Mcap*-CAH genome were transformed with 1–3 μg of the purified PCR cassette using lithium acetate, single-stranded carrier DNA and polyethylene glycol (32).

### Screening of yeast recombinants

Total DNA was extracted from the yeast recombinants (21,29) for screening by PCR, multiplex PCR and pulsed-field gel electrophoresis (PFGE). The PCR primers (Supplementary Table S1) were positioned immediately upstream and downstream of Mcap0476 and Mcap0613 to check for their respective replacement by the CORE/TANDEM cassettes (Figure 2A) and the *URA3* cassette (Figure 2B).

For multiplex PCR, the Multiplex PCR Qiagen kit was used with 50–100 ng of yeast DNA template and 10 pairs of primers (Supplementary Table S1) at 0.5 μM. The primers were distributed over the *Mcap*-CAH genome to facilitate screening for undesired genome rearrangements (Supplementary Figures S2 and S3).

Subsequently, the size of mycoplasma genomes was determined by PFGE. Yeast agarose plugs were prepared as previously described (21). Yeast DNA was fragmented with a cocktail of restriction enzymes (AsiSI, FseI and RsrII) that have multiple recognition sites in yeast chromosomes and none in *Mcap.* The yeast DNA fragments were electrophoresed from the agarose plugs. Circular *Mcap* DNA remained in the plugs and was specifically restricted with BssHII or PspXI. PFGE was performed on 1% agarose pulsed-field gel (Bio-Rad) with a contour-clamped homogeneous electric field (CHEF DR III; Bio-Rad). Pulse times were ramped from 60 to 120 s for 24 h at 6 V/cm and gels were stained with SYBR Gold.

### Transplantation and analysis of modified genomes in *Mcap* recipient cells

Recombinant *Mcap* genomes were released from the gel plugs and transplanted to *Mcap*-wt recipient cells cultured at 30°C in SOB(+) medium. Prior to transplantation, the donor DNA was methylated with *Mcap*-wt cellular extracts in order to protect it against *Mcap*-wt restriction enzymes (21). After transplantation, *Mcap* genomic DNA was extracted with the Wizard genomic DNA purification kit (Promega) prior to PCR reactions (Supplementary Figure S4).

### Complementation of the Δ*Mcap0476* mutant phenotype

Mcap0476 was amplified by PCR from *Mcap*-wt DNA using the primers trmFO1for (5′-ATTTAGATCTTTAAATATTTGTTCTTAATATTTTA) and trmFO1rev (5′-TATAAGATCTATGAATAA GAAAGTTAAAATTATTG) containing BglII sites (underlined) and was inserted into the same site in plasmid pPS3.1 (19) positioning the Mcap0476 coding sequence between the spiralin promoter (PS) and the fibril terminator (Tfb). The PS-Mcap0476-Tfb cassette was then amplified using the PCR primers Promo-Spi-EcoRI (5′-GATCGAATTCGATCCTCTAGAGTCGACCTGCAG) and Ter-fibril-EcoRI (5′-GATCGAATTCGCTTGCATG CCTGCAGTTCAGATC) containing EcoRI sites (underlined), and was inserted into the same site in plasmid pMYCO1 (20) to generate pMYCO1-Mcap0476 (Supplementary Figure S5), which contains a mycoplasma *oriC* and a tetracycline marker for selection. The Δ*Mcap0476* mutant was transformed (33) using 10 μg of plasmid. Plasmid DNA was isolated from tetracycline resistant clones using Wizard Plus SV Miniprep DNA Purification (Promega) and analyzed by restriction digestion with PstI. Total RNA was extracted from positives clones (Tri Reagent, Sigma) and Mcap0476 transcripts were detected by RT-PCR analysis (ThermoScript Reverse Transcriptase, Invitrogen) using the primer pair TrmFO1adir-3 (5′-GTTATATAGTTAGTTAGTG) and TrmFO1arev-3 (5′-GTTAGTTATATTGATGATG) giving a Mcap0476 cDNA fragment of 788 bp. Transcripts of Mcap0613 were detected with the primer pair TrmFO2dir2 (5′-GTTCATTTCACATTAAATTAG) and TrmFO2rev2 (5′-GAACTCGCATATTCTGATTC) producing a cDNA fragment of 688 bp.

### Analysis of RNA by matrix-assisted laser desorption-ionization mass spectrometry (MALDI-MS)

*Mycoplasma* cells (∼0.5 g) were washed in 100 ml of 50 mM Tris-HCl pH 7.5, 10 mM MgCl_2_, 100 mM NH_4_Cl, centrifuged and resuspended on ice in 2 ml of the same buffer. Cells were lyzed by sonication, and the debris was removed by centrifugation. Total cellular RNAs were extracted with phenol/chloroform, recovered by ethanol precipitation and dissolved in H_2_O.

**Figure 3.**
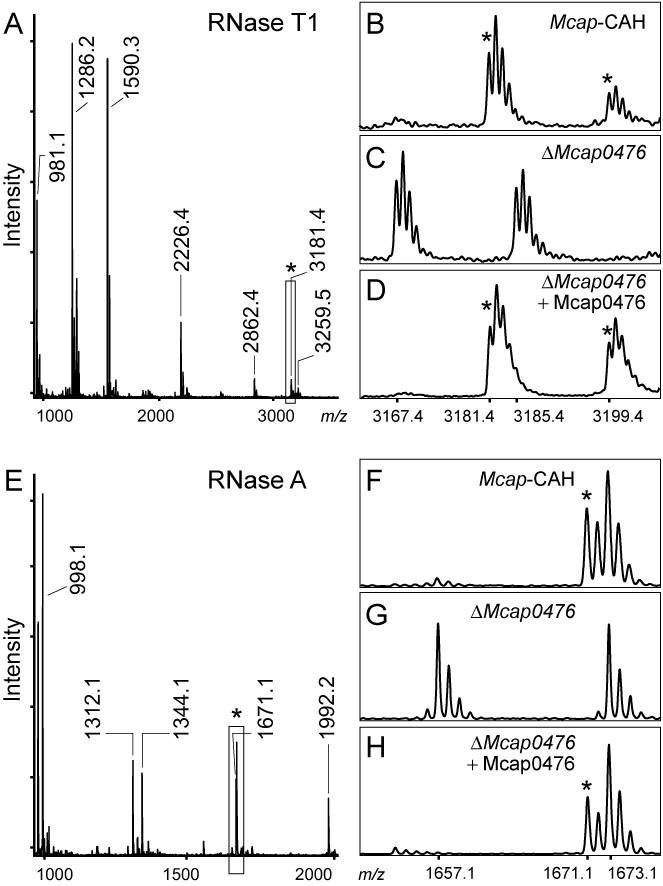
MALDI-MS spectra of the U1939 region from the *Mcap* strains. Peaks containing m^5^U1939 are indicated (*). (**A**) RNase T1 digestion products from the *Mcap* 23S rRNA sequence G1910-G1964. In the wild-type 23S rRNA from *Mcap*-CAH, nucleotide U1939 is present in the unique oligonucleotide AAA[m^5^U]UCCUUG>p with a single methyl group. This oligo (boxed) flies at *m/z* 3181 with a cyclic 2′,3′-phosphate, and at *m/z* 3199 with a fully hydrated linear 3′-phosphate. (**B**) Enlargement of this spectral region for *Mcap*-CAH and (**C**) the *ΔMcap0476* strain, where reduction in the oligonucleotide mass (*m/z* 3167 and 3185 with cyclic and linear phosphates) corresponds to the loss of a methyl group. (**D**) Methylation of the rRNA region was restored after complementation of the *ΔMcap0476* strain with an active copy of Mcap0476 expressed from a plasmid. (**E**) RNase A digestion of the wild-type rRNA where U1939 is in the fragment GAAA[m^5^U]p at *m/z* 1671, and can be seen in the enlargement (**F**) to be adjacent to the unmodified G1930-U1934 peak GGAAUp at *m/z* 1673 (all fragments with linear 3′-phosphates). (**G**) In the corresponding spectrum from the *ΔMcap0476* rRNA, U1939 lacks a methyl group and flies at *m/z* 1657, while the *m/z* 1673 fragment remains unaltered. (**H**) Transformation of the *ΔMcap0476* strain with a plasmid-encoded copy of Mcap0476 restores rRNA methylation. The single *ΔMcap0613* knockout strain showed the same methylation pattern as the wild-type strain in panels (B) and (F), whereas the *ΔMcap0476/ΔMcap0613* double knockout lacked the U1939 methylation as in panels (C) and (G) (not shown). Other peaks of interest: the RNase A sequence AA[Cm]GGUp at *m/z* 1992 confirms Cm1920; the RNase T1 sequence AAC[m^3^Ψ]AUAA[Cm]G>p at *m/z* 3259 contains m^3^Ψ1915 and Cm1920; and the RNase T1 sequence ACCCG>p at *m/z* 1590 shows that C1962 is unmodified. The empirical and theoretical masses match within 0.2 Da; all values are for ^12^C monoisotopic masses. The multiple tops in the enlargements reflect the natural ^12^C/^13^C distribution.

Total RNA extracts from the wild-type, *ΔMcap0476*, *ΔMcap0613*, *ΔMcap0476/ΔMcap0613* and Mcap0476-complemented *ΔMcap0476* strains were analyzed within the 23S rRNA regions previously shown to contain m^5^U methylations in other organisms. In each case, 100 pmol of total RNA was hybridized to 500 pmol of the 48-mer deoxyoligonucleotide, 5′-GCCACAAGTCATCCAAAGTCTTTTCAACGAATACTGGTTCGGTCCTCC, complementary to the sequence G725-C772 in domain II of the 23S rRNA (Figure 3), or to the 55-mer 5′-CGGGTCAGAATTTACCTGACAAGGAATTTCGCTACCTTAGGACCGTTATAGTTAC, complementary to the sequence G1910-G1964 within domain IV of the 23S rRNA (Figure 3). The exposed regions within the RNAs were digested away with mung bean nuclease and RNase A, and the sequences protected by hybridization were separated on gels (34,35). The rRNA fragments of ∼48 and 55 nucleotides were extracted and digested with either RNase A or RNase T1 in aqueous solution containing 3-hydroxypicolinic acid and analyzed by MALDI-MS (Ultraflextreme, Bruker Daltronics) recording in reflector and positive ion mode (36). Spectra were processed using the program Flexanalysis (BrukerDaltonics).

### HPLC analyses of tRNAs

The tRNA fraction was isolated from the total RNA mixture using Nucleobond^®^ RNA/DNA 400 kits (Macherey-Nagel). Bulk tRNAs were digested to completion to form nucleosides (37), which were subjected to reverse-phase chromatography on a Agilent Technologies 1200 series HPLC with a Phenomenex Luna C18 column (2 × 250 mm, 5 μm particles, 100 Å pores). Nucleosides were eluted essentially as described previously (37) with 40 mM ammonium acetate pH 6 and introducing a linear gradient of acetonitrile after 2 min that rose to 10% at 27 min, then to 24% at 37 min, and finally to 40% acetonitrile. The column flow rate was set to 250 μl/min at 40°C and eluents were detected at 260 nm.

## RESULTS

### *In silico* identification of putative *Mcap* m^5^U methyltransferases

Our initial finding of m^5^U in *Mcap* 23S rRNA was clearly at odds with bioinformatics data showing an absence of AdoMet-dependent COG2265 enzymes that add this modification in other bacteria (15). A search for candidates responsible for m^5^U catalysis in *Mcap* revealed Mcap0476 and Mcap0613, two *trmFO*-like homologs belonging to COG1206. These candidates were taken into consideration with the proviso that previously characterized TrmFO homologs are specific for the m^5^U54 methylation in tRNA (10–14,38). Alignment of TrmFO sequences (Supplementary Figure S1A) indicated that the Mcap0476 protein has the highest identity (47%) with its *B. subtilis* counterpart, although this level of identity is significantly lower than between TrmFO orthologs such as *B. subtilis* and *Thermus thermophilus* (69% identical) that are known to methylate tRNA (Supplementary Figure S1B).

The nearest known relative of the Mcap0613 protein is presently the *B. subtilis* TrmFO with 38% identity, a value that is not significantly greater than between Mcap0476 and Mcap0613 (39%). The two cysteines Cys53 and Cys226, which have been shown to play major roles in catalysis by the *B. subtilis* enzyme (38,39), are present in the Mcap0476 protein but are absent in Mcap0613 (Supplementary Figure S1A). The Mcap0476 protein also possesses most of the conserved residues that are important for the activity of *T. thermophilus* TrmFO (13), however, these two proteins differ in their C-terminal regions where interaction with their respective RNA targets is presumed to occur. Some conserved residues have been retained in Mcap0613, although there is significant sequence divergence from other TrmFO homologs including Mcap0476 (Supplementary Figure S1A).

Tertiary structure modeling for the Mcap0476 and Mcap0613 proteins (Supplementary Figure S1C) produced close matches to the *T. thermophilus* TrmFO crystal structure (13). Superimposition of the Mcap0476 and Mcap0613 models onto the *T. thermophilus* TrmFO structure showed essentially the same fold, where both *Mcap* proteins lacked an AdoMet binding site (5), but possessed structures resembling the folate and FAD binding regions seen in TrmFO (13).

### Strategy for investigating Mcap0476 and Mcap0613 function

The genome in *Mcap* was equipped with genetic elements for stable replication and selection in yeast and with an antibiotic resistance marker for selection in mycoplasma (25). The resultant *Mcap*-CAH genome (Supplementary Figure S2) was transferred into yeast cells, and one clone (W303a-*Mcap*-CAH) was selected to inactivate Mcap0476 and Mcap0613 individually and in combination using two knockout strategies (Figures 1C and 2). Both strategies involve transformation of W303a-*Mcap*-CAH with PCR-generated cassettes containing portions of the targeted sequences to effect gene excision via homologous recombination.

The first of these strategies (TREC) (30) replaces the targeted gene (in this case Mcap0476) with mutagenesis cassettes, which are later excised generating a seamless deletion of the gene (Figure 2A). The mutagenesis cassettes replaced Mcap0476 in one yeast transformant out of seven tested (clone 3). Upon subsequent activation of the I-SceI endonuclease, cassette excision occurred in one subclone out of eight tested (*ΔMcap0476* subclone 3.2). PCR analyses confirmed that Mcap0476 together with the nuclease/marker genes had been seamlessly deleted (Figure 2A).

A second approach was used to inactivate Mcap0613 by direct replacement with *URA3* to create a single knockout in the *Mcap*-CAH genome (Figure 2B) and a double knockout in the *ΔMcap0476* genome (Figure 2C). This approach gave a higher frequency of desired recombinants (9 out of 13 tested for the *ΔMcap0613* single knockout; and 12 out of 16 for the *ΔMcap0476*/*ΔMcap0613* double knockout). The *URA3* gene remains in the mycoplasma genome, and thus this technique is suitable for inactivating the last gene after a series of markerless knockouts generated by iterative rounds of TREC.

At each stage, the yeast recombinants were screened by PCR (Figure 2), and the structure and size of the *Mcap* chromosomes were checked by multiplex PCR and pulsed-field gel electrophoresis (Supplementary Figure S3). Mutant *Mcap* chromosomes were then extracted from the yeast host in agarose gel plugs, and the *Mcap* DNA was methylated *in vitro* to avoid restriction when transplanted back into *Mcap* recipient cells. Selection with puromycin ensured that the wild-type genome was replaced by the Δ*Mcap0476*, Δ*Mcap0613* or Δ*Mcap0476*/Δ*Mcap0613* genomes. After verifying the genome structures (Supplementary Figure S4), the RNAs of positive clones were analyzed for modification.

### Mcap0476 and Mcap0613 products do not methylate tRNA

Bulk tRNA was purified from the *Mcap*-CAH and knockout strains, and nucleotide modifications were tested by HPLC and matrix-assisted laser desorption/ionization mass spectrometry (MALDI-MS). HPLC analysis of wild-type tRNA nucleosides derived from the *Mcap*-CAH strain showed a complete absence of m^5^U (Supplementary Figure S6) consistent with earlier reports for this species (22–24). Analysis by MS (Supplementary Figure S7) revealed no obvious differences in the modification patterns of the tRNAs from *Mcap*-CAH and the knockout strains.

### Mcap0476 encodes the methyltransferase RlmFO that adds the m^5^U1939 rRNA modification

The rRNAs from the *Mcap* strains were analyzed by MALDI-MS. Digestion of the 23S rRNA domain IV sequence with RNase T1 produced a unique decamer of nucleotides 1936–1945 AAAUUCCUUG>p, containing U1939 (underlined). The wild-type decamer from the *Mcap*-CAH strain was recorded at *m/z* 3181 (Figure 3A) and corresponded to the expected nucleotide sequence plus a single methyl group. RNase A digestion of this same rRNA region produced an overlapping fragment of nucleotides 1935–1939 GAAAUp at *m/z* 1671 (Figure 3E) also containing the methyl group, which was localized to nucleotide U1939. The methyl group interfered with neither RNase A digestion (Figure 3E) nor with reverse transcriptase extension (not shown), which respectively ruled out its attachment to the 2′-*O*-ribose or the 3-position of the base (35,40), and was consistent with methylation at the C5-position of U1939.

The rRNAs from the *ΔMcap0476*, *ΔMcap0613* and *ΔMcap0476/ΔMcap0613* derivatives of the *Mcap*-CAH strain were examined in a similar manner. The *ΔMcap0613* strain showed the wild-type methylation pattern identical to the *Mcap*-CAH strain, whereas rRNAs from the single *ΔMcap0476* and double *ΔMcap0476/ΔMcap0613* knockout strains produced an RNase T1 AAAUUCCUUG>p fragment at *m/z* 3167 (Figure 3C) with an overlapping RNase A fragment at *m/z* 1657 (Figure 3G). These observations indicate that inactivation of Mcap0476 results in loss of methylation at 23S rRNA nucleotide U1939. The m^5^U1939 modification was recovered in the *ΔMcap0476* strain after transformation with a plasmid encoding an active copy of Mcap0476 (Figure 3D and F).

**Figure 4.**
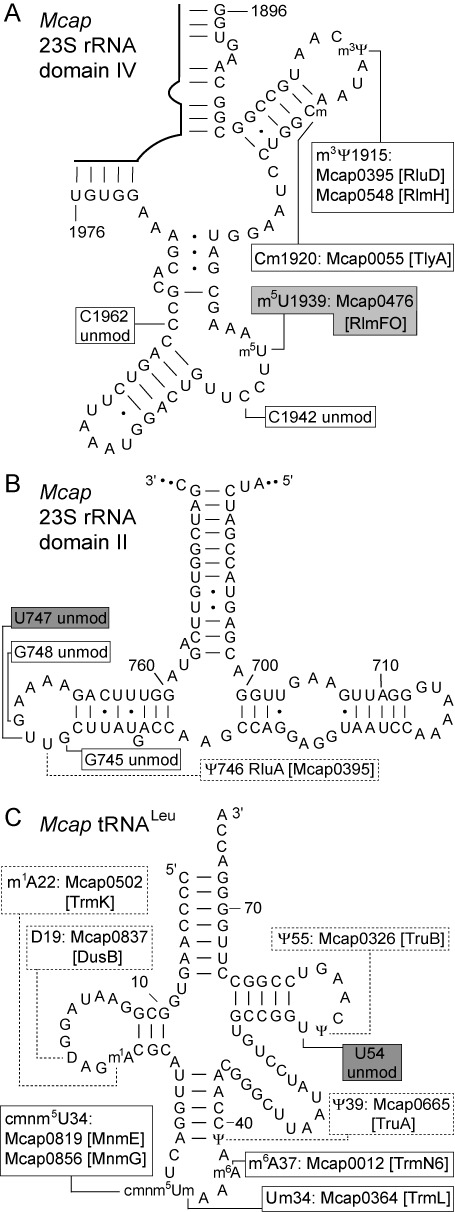
*Mcap* RNA secondary structures showing the modification sites. The positions of m^5^U modification known from other organisms are indicated (gray boxes). (**A**) Region of *Mcap* 23S rRNA domain IV with the RlmFO (Mcap0476) methyltransferase product m^5^U1939. Modifications at other nucleotides were evident during this study, and are indicated (white boxes) together with the putative *Mcap* enzymes (15). Nucleotides 1942 and 1962 are unmodified in *Mcap* rRNA (unmod) whereas m^5^C is found at these positions in some bacteria (49,50). (**B**) In certain bacteria, methylation occurs at the conserved nucleotides G745, U747 and/or G748 (3,51,52). There was no methylation in this region of *Mcap* 23S rRNA domain II (Supplementary Figure S8) and, consistently, *Mcap* possesses none of the orthologous methyltransferases. (**C**) Structure of the *Mcap* tRNA^Leu^ UAA isoacceptor with nucleotide modifications (24). We confirmed the absence of modification at U54 using HPLC (Supplementary Figure S6), and the anticodon loop modifications were consistent with our MS (Supplementary Figure S7) and bioinformatics analyses. Modifications in the dashed boxes were not tested empirically in the present study, but were supported by bioinformatics.

Some bacterial 23S rRNAs also have m^5^U at nucleotide 747 (3,6), and this modification was shown to be absent in *Mcap* (Supplementary Figure S8). Other modifications were observed during the analysis of *Mcap* 23S rRNA including methylation at m^3^Ψ1915 and the 2′-*O*-ribose of C1920, while some modifications found in other bacteria are missing in *Mcap* (Figure 4). These empirical findings correlated well with bioinformatics analyses showing the presence or absence of *Mcap* gene orthologs for known RNA modification enzymes (15).

## DISCUSSION

Methylation of uridine to form ribothymidine (m^5^U) is a widespread modification that contributes to the functional fine-tuning of tRNAs and rRNAs in all three domains of life (1–3). With the exception of the tRNA m^5^U54 modification added by TrmFO in a subset of bacteria (10–14,38), these methylations are catalyzed by enzymes that require AdoMet as the methyl donor. In fact, all other types of methylation previously characterized in rRNAs are added by AdoMet-dependent enzymes (5). The data presented here shows that an alternative rRNA modification mechanism exists in the minimalist mollicutes bacterium, *Mcap*. The methyltransferase encoded by Mcap0476 adds the m^5^U1939 modification in *Mcap* 23S rRNA and is structurally similar to the TrmFO enzymes shown to bind *N*^5^,*N*^10^-methylenetetrahydrofolate and FAD, while lacking motifs required for AdoMet interaction (Supplementary Figure S1). The Mcap0476 enzyme is the first rRNA-targeting methyltransferase seen to be dependent on folate/FAD for its activity and has thus been renamed RlmFO (rRNA large subunit methyltransferase, folate-dependent).

The m^5^U1939 modification is found in the 23S rRNAs of other bacteria (7,9) where it is added by AdoMet-dependent methyltransferases. Thus, two mechanistically distinct types of enzymes have evolved independently to methylate the same rRNA nucleotide. A similar case has been described for the AdoMet-dependent TrmA (8) and folate-dependent TrmFO enzymes (10), the functions of which have converged during evolution to catalyze the m^5^U54 modification in tRNA. This raises the question as to how specific recognition of a single nucleotide target developed in these different types of enzyme. The AdoMet-dependent methyltransferases TrmA and RlmC/D (6,7,41), which respectively target U54 in tRNA and U745/U1939 in 23S rRNA, are believed to have arisen from duplication of an ancestral COG2265 gene for a multi-site specific enzyme (42) followed by target-specialization of paralogs. This idea was recently supported by characterization of the *B. subtilis* enzyme RlmCD (9) that possesses dual-site specificity for U747 and U1939 in 23S rRNA. Thus, it is feasible that ancestral versions of RlmCD-type enzymes were able to accommodate a larger range of RNA targets into their active site.

It is not immediately obvious how enzymes such as RlmCD (and ancestors) might have limited their target selection to two (or a few) specific uridines, especially when these nucleotides are displayed in RNA regions with apparently dissimilar primary and secondary structures (Figure 4). Some clarification comes from crystallographic models, which reveal how the U54 and U1939 RNA regions are amenable to being refolded into similar conformations that enable the target uridine to be flipped into the active site of the enzyme (43). Structural malleability of the tRNA and rRNA targets is possibly also required by the folate-dependent TrmFO and RlmFO enzymes that modify these same nucleotides. It can thus be envisaged that a progenitor folate-dependent m^5^U methyltransferase was multi-site specific, and evolved in a manner comparable to RlmCD with gene duplication and specialization amongst paralogs leading to the site-specific TrmFO and RlmFO variants seen today. One prediction from this model would be that dual- or multi-site versions of TrmFO/RlmFO might still exist. A prospective search for multi-site enzymes might best be directed to the presently surviving mycoplasmas and related genera with minimal genomes. Functional genomics study of such organisms, which were previously regarded as intractable, has now become feasible using synthetic biology tools such as those developed to generate clean single and multiple gene deletions in *Mcap*.

Considering the phylogenetic conservation of the m^5^U modifications and the evolution of more than one mechanism to maintain them, it might be expected that their loss would cause a severe phenotypic disadvantage. However, no such effect was observed comparing the growth of *Mcap*-CAH and *ΔMcap0476* strains, which grew in liquid culture with doubling times of 116 and 118 min, respectively. Similar observations had also been made for *E. coli* where there was no marked growth rate reduction after inactivation of its m^5^U1939 methyltransferase RlmD (44), or even after inactivating RlmD together with its two m^5^U COG2265 paralogs (9). Likewise, a benign effect was seen in *B. subtilis* after inactivation of the dual-site m^5^U methyltransferase RlmCD (9). There are precedents at other rRNA and tRNA nucleotides where the loss of highly conserved modifications are accompanied by relatively minor phenotypic changes, and such effects are perhaps explained by the different growth conditions bacteria experience in the wild compared to the laboratory (45,46).

The lack of the otherwise universally conserved m^5^U54 modification in *Mcap* tRNAs ((22–24) and Supplementary Figure S6) might suggest that the *Mcap* translation apparatus has undergone compensatory changes to cope with this deficiency. We checked whether such changes might have occurred in modified regions of tRNAs, and in particular those with cmnm^5^U34 formed by carboxymethylaminomethylation of the anticodon wobble base. This modification is added in other bacteria by the MnmE/MnmG complex using FAD and methylene-tetrahydrofolate (47) and, although orthologous enzymes in *Mcap* are encoded by Mcap0819 and Mcap0856 (15), similarities in the cofactor binding domains led us to check for a possible role for the orphan TrmFO homolog Mcap0613. MS fragments containing cmnm^5^U34 were unambiguously identified in tRNA^Leu^ (Supplementary Figure S7) and tRNA^Trp^ from Mcap-CAH, and were also clearly seen in tRNAs from the *ΔMcap0476* and *ΔMcap0613* single knockouts and the *ΔMcap0476/ΔMcap0613* double knockout. There was thus no indication of Mcap0613 (or Mcap0476) being connected with the formation of cmnm^5^U or any of the nearby anticodon modifications. We have similarly ruled out involvement of Mcap0613 in m^5^U1939 methylation (Figure 3) and even though the Mcap0613 gene is clearly transcribed in *Mcap* (Supplementary Figure S5E), we have yet to find a function for the Mcap0613 enzyme. Taken together with its degenerate sequence motifs (Supplementary Figure S1A), there is no evidence at present that the Mcap0613 enzyme plays a role in tRNA or rRNA modification.

In conclusion, the function of the RlmFO (Mcap0476) enzyme has been defined by application of a synthetic biology approach, and is the first example of a folate-dependent flavoprotein that specifically methylates rRNA. Mycoplasmas and related genera represent attractive models for exploring RNA modifying mechanisms and determining the minimal set of RNA modifying enzymes necessary to maintain protein synthesis (48).

## SUPPLEMENTARY DATA

Supplementary data are available at NAR Online.

SUPPLEMENTARY DATA
