# Supplementary material for: The flavoprotein Mcap0476 (RlmFO) catalyzes m5U1939 modification in Mycoplasma capricolum 23S rRNA
Source: Nucleic Acids Res. 2014 Jun 17;42(12):8073–82. doi: 10.1093/nar/gku518 (PMC4081110; doi:10.1093/nar/gku518)
Supplement: SUPPLEMENTARY DATA [file supp_42_12_8073__index.html]

The flavoprotein Mcap0476 (RlmFO) catalyzes m5U1939 modification in Mycoplasma capricolum 23S rRNA — SUPPLEMENTARY DATA 

# The flavoprotein Mcap0476 (RlmFO) catalyzes m5U1939 modification in *Mycoplasma capricolum* 23S rRNA

## SUPPLEMENTARY DATA

**Files in this Data Supplement:**

- SUPPLEMENTARY DATA
