# Supplementary material for: The flavoprotein Mcap0476 (RlmFO) catalyzes m5U1939 modification in Mycoplasma capricolum 23S rRNA
Source: Nucleic Acids Res. 2014 Jun 17;42(12):8073–82. doi: 10.1093/nar/gku518 (PMC4081110; doi:10.1093/nar/gku518)
Supplement: SUPPLEMENTARY DATA [file supp_gku518_nar-00726-r-2014-File006.pdf]

Supplementary Data

The flavoprotein Mcap0476 (RlmFO) catalyzes m^5U1939 modification in *Mycoplasma capricolum* 23S rRNA

Carole Lartigue,[1,2*] Anne Lebaudy,[1,2*] Alain Blanchard,[1,2]
Basma El Yacoubi,[3] Simon Rose,[4]
Henri Grosjean[5§] & Stephen Douthwaite[4§]

[1] INRA, UMR 1332 de Biologie du Fruit et Pathologie, F-33140 Villenave d'Ornon, France.

[2] Univ. Bordeaux, UMR 1332 de Biologie du Fruit et Pathologie, F-33140 Villenave d'Ornon, France.

[3] Dept. Microbiology and Cell Science, University of Florida, USA.

[4] Dept. Biochem. & Mol. Biol., University of Southern Denmark, DK-5230 Odense M, Denmark.

[5] Centre de Génétique Moléculaire, UPR3404, CNRS, Associée à l'Université Paris Sud 11, FRC 3115, F-91190 Gif-sur-Yvette, France.

* Contributed equally to this work

[§] Correspondence: Henri Grosjean, henri.grosjean@cgm.cnrs-gif.fr; and Stephen Douthwaite srd@bmb.sdu.dk

This file includes
Supplementary Figures 1 to 8
Supplementary Table 1
References

A

```
                                                                                Cys53
Mcap0476      MN-KKVKIIGAGLAGCEAAYFLANNDIQVELYEVKTLIKNEVQKTNNFAELVCSNTFRSQSLLNAAGILKAEMRRLNSLVIKIADSCKIDGDDALAVDRE
Mcap0613      MK--TIRIIGAGLSGCEAAYYLLKKGYFVELYEIKTIKKNPIQHYDYFCELAYSDSFRSTDLNTSVGTLKKELELLDSLIIKAARYASINQNNELVVNRI
Tther_TrmFO   ME--RVNVVGAGLAGSEAAWTLLRLGVPVRLFEMRPKRMTPAHGTDRFAEIVCSNSLGGEGETNAKGLLQAEMRRAGSLVMEAADLARVPAGGALAVDRE
Bsubt_TrmFO   MNQQTVNVIGAGLAGSEAAWQLAKRGIQVKLYEMRPVKQTPAHHTDKFAELVCSNSLRSNTLANAVGVLKEEMRALDSAIIAAADECSVPAGGALAVDRH
Aaeol_TrmFO   MK-EKVIVIGGGLAGSEAAWRLANEGHRVILYEMRPKKMTPAHKTGNLAELVCSNTLGGLELTTGAGLLKAEMQKLGSLVIEAAKVARVPAGGALGVDRK
Tmari_TrmFO   M---IVNVIGAGLAGSEVAYNLGKRGIRVRLFEMRPKKMTEVHKTGYFAELVCSNSLKSEDITNAEGLLKAEMRLMGSITLEAAEKARVPSGKALAVDRN
              *    : ::*.**:*.*.*.*: *  .  .   *  *:*::.    .   :   . :.*:. *::: .     .. * *: *:.  .*  :  *   . :  .   * *:*

Mcap0476      DFSKKLTDVIKNHPNITIIEQNVSYIDDENDLTLIATGPLTTNELKEDIQRLIGKQKLFFMDASAPIITKDSIDFNKVYYSGRHKQG--KYICCPLNEQE
Mcap0613      EFSKYITNYLKTFNNLKIIEQEYLNIDL-NIPTIIAIGPISTPNFLTNLKKLINKENLKLFDTVEPTILKQSINMDICYSLD---NN-LNYLYCDLNKEQ
Tther_TrmFO   EFSGYITERLTGHPLLEVVREEVREIPP--GITVLATGPLTSEALAEALKRRFGDHFLAYYDAASPIVLYESIDLTKCFRAGRYGQS-ADYLNCPMTEEE
Bsubt_TrmFO   EFAASVTNRVKNHPNVTVINEEVTEIPE--GPTIIATGPLTSESLSAQLKELTGEDYLYFYDAAAPIVEKDSLDMDKVYLKSRYDKGEAAYLNCPMTEEE
Aaeol_TrmFO   IFSEYITEKIESHPNITLIREEVKEIPE-DEVVVIATGPLTSDALSEKIKELVGYDTLYFYDAIAPIVEAESVDFSKGFWGSRYGKGGDDYFNCVLTEEE
gi|351678177  IFAKEVTEVIERLESVEIIREEVTEFDPEEGIWVVATGPATSDGLLPFLKKLLGDDLLFFFDAVSPIVTFESIDMECAFWGDRFGKG-KDYINCPLTKEE
               *:   :*: :      : ::.::    :        ::* ** ::  :    ::.   . . *    *:   * :  :*:::    :   .    :.    *: * :.:::

                                           Cys226
Mcap0476      FNKFVDDLVNAEQVQLKEFEKSIFFK--GCQPIEQLAKTSKKLLLKGPMSPNNLLDQ-NNQQPYAVVQLRQDDAKDSLYNMVGFQTNLKWPEQKRVFQTI
Mcap0613      FEKFYNALISAKTFNSPLKNEIELLEKNNYFSIESLAKNKQEFI--NHFKPI-------NNNAYITITLKKDSVINNLYTIVNFQTNLMWNEQLKVFSLI
Tther_TrmFO   YRRFHQALLEAQRHTPHDWEKLEFFE--ACVPVEELARRGYQTLLFGPMKPVGLVDPRTGKEPFAVVQLRQEDKAGRMWSLVGFQTGLKWPEQKRLIQMI
Bsubt_TrmFO   FDRFHEALTSAETVPLKEFEKEIFFE--GCMPIEVMAKRGKKTMLFGPMKPVGLEHPVTGKRPYAVVQLRQDDAAGTLYNIVGFQTHLKWGDQKEVLKLI
Aaeol_TrmFO   YKKFYEELLKAEKVKPKDFEKAVHFE--GCLPIEEMAERGYKTLLFGPMKPVGLVDPRTGKEPFAVVQLRKENKEGTLLSLVGFQTKLTYKEQKRVFRLI
Tmari_TrmFO   YEEFWKALVEAEVIEMEDFDRKLLFE--RCQPIEEIARSGKDALRYGPLRPTGLVDPRTGKEPYAVVQLRREDKEGRFYSLVGFQTRLKWSEQKRVLRKI
              : .* . * .*:         :.    ::      .:* :*.     . :    . :  *          .:..: .: *:::.   . :  .:*.*** *  :  :* .::   *

Mcap0476      PGLEKAKIVRYGVMHKNYYINSPKILNFKLQVIRKKNVFFAGQITGVEGYIESASSGIWAAINILAFINNKKIKPLPNTTILGALTNYITNSKIYSLKPM
Mcap0613      PGLENLKIMKYGVMHKNNYINTKKLLNLGVQLKTNKNIFFAGQIIGVDGYVESVCSGLISAINLDRYLNNKKMIIPNKNSTIGSLYNYLLKT-DSNFSPM
Tther_TrmFO   PGLENAEIVRYGVMHRNTYLNAPRLLGETLEFREAEGLYAAGVLAGVEGYLESAATGFLAGLNAARKALGLPPVAPPEESMLGGLVRYLATANPEGFQPM
Bsubt_TrmFO   PGLENVEIVRYGVMHRNTFINSPSLLKPTYQFKNRSDLFFAGQMTGVEGYVESAASGLVAGINAAKLVLGEELVIFPQETAIGSMAHYITTTNQKNFQPM
Aaeol_TrmFO   PCLRNAVFVRLGSMHRNTFIQSNKVLTHYLNLKKKENIFFAGQITGVEGYVASSATGILAGINAGRLARGEKPLKAPTETMLGALVNYIVTK-EGELQPM
Tmari_TrmFO   PCLRNAEIVRYGVMHRNVYINSPKLLDIFFRLKKHPNIFFAGQITGVEGYMESAASGIYVAYNVHRILKGLSPLKLPEETMMGALFSYIIEKVEGDLKPM
              * *.:   ::: * **:* ::::   :*     ..     .:: ** : **:**: * .:*:   . *        .           : :*.:   *:         :.**
```

```
Mcap0476     KCNLGILEQENKY-QSNDKFYS--YNNSKNSLENYIEQLNKI----LRTNI
Mcap0613     RINWALVDMIGGF-ELSDNSKKIYSKRAIKLIKQYLKKI----------NT
Tther_TrmFO  YANWGLVPPVEGRMGKKE-KRQAMYRRGLEAFSAWLSGLNPPLPRPEAALV
Bsubt_TrmFO  NANFGLLKELPVKIKNKKERNEQYANRAIETIQTIS------------KTI
Aaeol_TrmFO  NPVFGLLPPLEKKVRDKKKRKELMAKRALETMEKWIKENNL-VPEGLVKVC
Tmari_TrmFO  YANFGLLPPLKVRVKDKFEKRKKLAERAIETMKKFLEEN----------PW
                 .::         . :  .   ... : :.
```

B

	Mcap0476	Mcap0613	Ttherm	Bsub	Aaeol	Tmari
Mcap0476		39.0	38.4	46.5	43.4	44.0
Mcap0613	59.4		28.4	37.7	29.4	32.7
Ttherm	60.0	50.4		50.9	52.7	51.3
Bsub	67.3	56.7	69.0		52.9	49.3
Aaeol	64.2	51.7	69.2	67.6		54.8
Tmari	63.9	54.9	67.3	68.6	72.3	

C

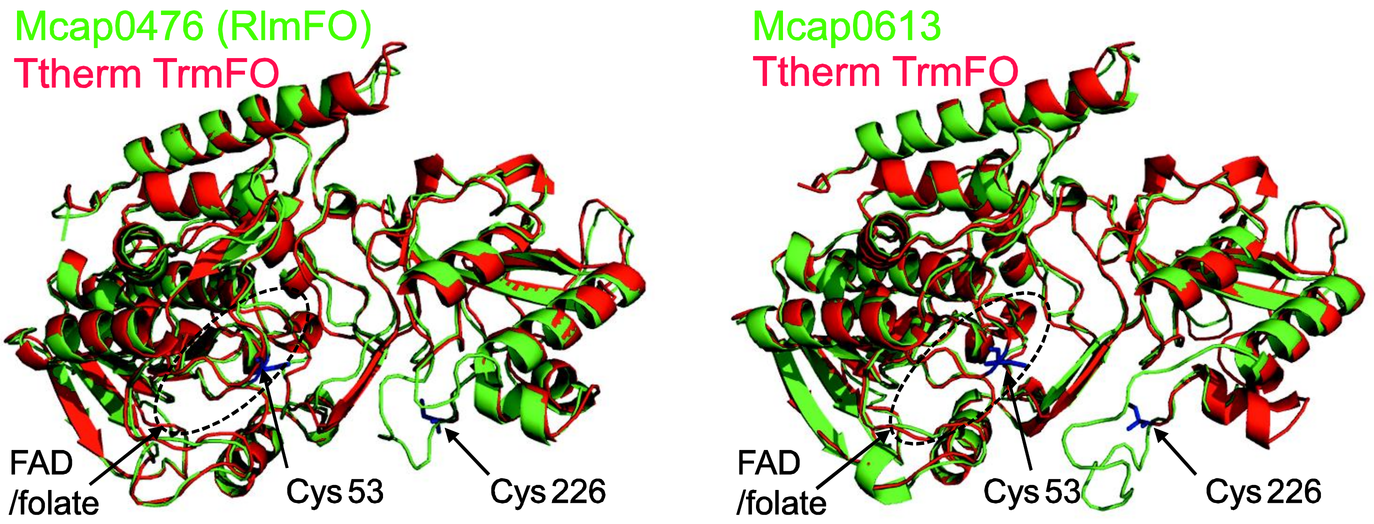

Supplementary Figure 1. Comparison of TrmFO-like proteins.

(**A**) Multiple TrmFO sequence alignment generated using ClustalW. TrmFO sequences from *T. thermophilus* (Tther_TrmFO), *B. subtilis* (Bsubt_TrmFO; the numbering system for this protein is used here), *Aquifex aeolicus* (Aaeol_TrmFO) and *Thermotoga maritima* (Tmari_TrmFO) are aligned against Mcap0476 and Mcap0613 proteins. The highlighted residues are thought to be involved in the binding of FAD (red), folate (green), GSH (orange) (1,2), tRNA (blue), and the formation of the domain interface (purple). The signature motif for the GSH reductase family (brown highlight) is shown at the N-terminus. Identical residues (*), conserved substitutions (:), and semi-conserved substitutions (.) are indicated under the sequences. Cys53 (yellow highlight) and Cys226 (green highlight) have been reported to play a major catalytic role in *B. subtilis* TrmFO (2).

(**B**) Identity and similarity between TrmFO-like sequences are indicated respectively as percentages above and below the diagonal, and were calculated from pairwise alignments using the EMBOSS Needle program. This program is available on the EBI web server: http://www.ebi.ac.uk/Tools/services/web/toolform.ebi?tool=emboss_needle&context=protein and uses the Needleman-Wunsch alignment algorithm. Parameters: Matrix, EBLOSUM62; Gap open, 10.0; Gap extend, 0.5; End Gap Penalty, false; End Gap Open Penalty, 10.0; End Gap Extension Penalty, 0.5. Abbreviations as in the sequence alignment.

(**C**) Structures for the Mcap0476 (RlmFO) and Mcap0613 proteins were calculated using I-TASSER (3). The closest published match to these models is the crystal structure (PDB 3G5R) of Tther TrmFO (1). The I-TASSER RlmFO and Mcap0613 models (green) are shown superimposed onto Tther TrmFO (red), and can be seen to have essentially the same fold. Both *Mcap* proteins lack an AdoMet binding site (4), but possess structures resembling the N^5, N^{10}-methylenetetrahydrofolate (folate) and FAD binding regions seen in Tther TrmFO (dashed ellipse). Modelling FAD into the structures using I-TASSER gave a good fit in the TrmFO, RlmFO and Mcap0613 proteins. Folate could be easily docked into the TrmFO structure, although docking of folate into the corresponding regions of the RlmFO and Mcap0613 proteins would require some structural rearrangement. The estimated accuracy of the model for RlmFO: confidence (C) score 1.32, template modelling (TM) score 0.90 ± 0.06. For Mcap0613: C-score 1.50; TM-score 0.92±0.06. An alternative modelling approach using Phyre2 (5) gave essentially the same folds for the RlmFO and Mcap0613 proteins (not shown). The catalytically important residues Cys53 and Cys226 (*B. subtilis* TrmFO numbering) are present in both Tther TrmFO and RlmFO, but absent in Mcap0613. These cysteines are 20 Å apart, and it has been suggested for TrmFO that dimerization occurs upon tRNA binding bringing Cys53 and Cys226 of the two monomer units into close proximity (2).

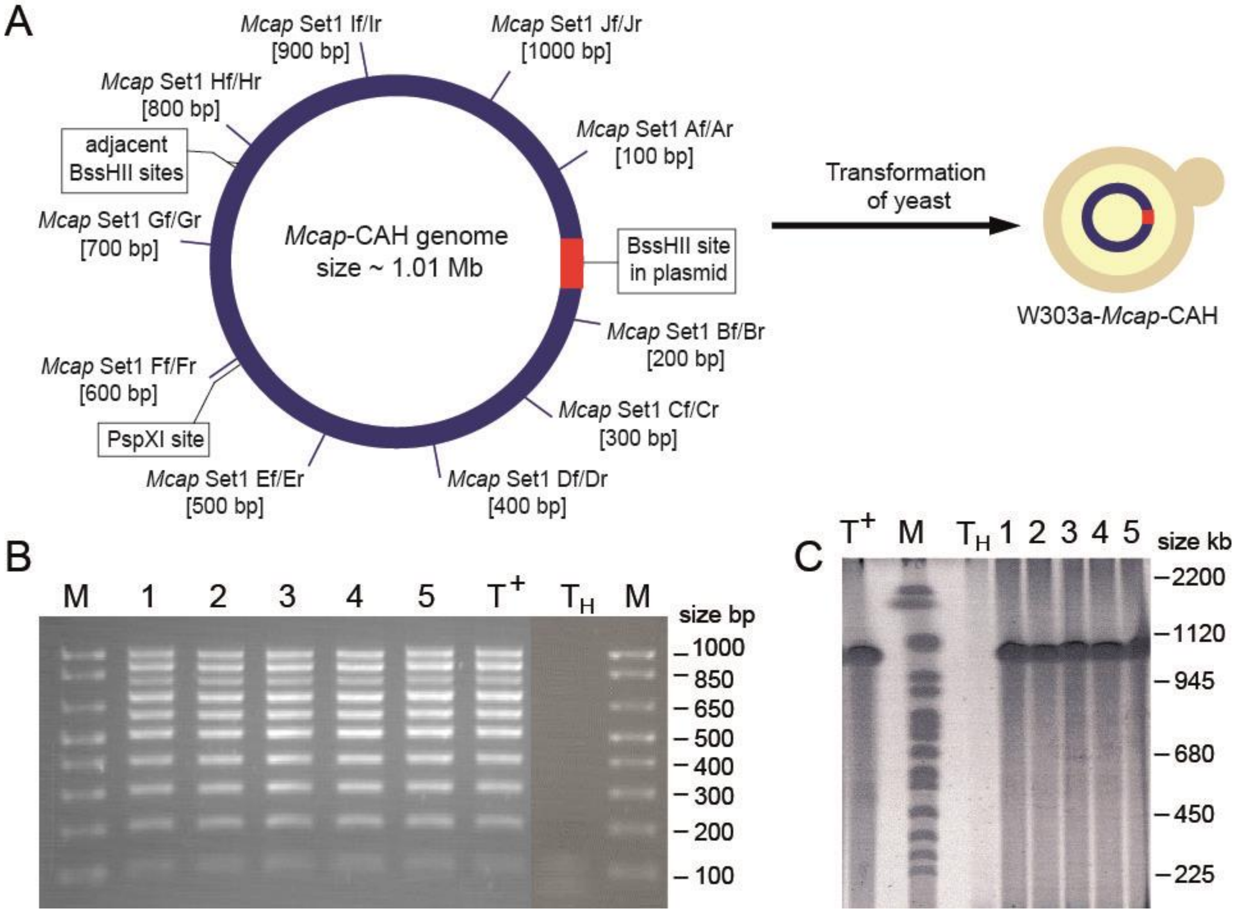

Supplementary Figure 2. Cloning of the *Mcap*-CAH genome in yeast. (**A**) *Mcap*-CAH was obtained by recombination of the plasmid pSD4-puro-CAH-Δ*Mcap0050* (red rectangle) into the *Mcap*-wt chromosome providing the elements for selection in yeast and mycoplasma (6). The map of the *Mcap*-CAH genome indicates the relative positions of pSD4-puro-CAH-Δ*Mcap0050*, PCR priming sites and BssHII and PspXI restriction sites. The engineered genome was isolated in an intact state from *Mcap*-CAH cells and transferred into yeast spheroplasts to form the yeast clone W303a-Mcap-CAH, where the *Mcap*-CAH genome was maintained as a centromeric plasmid. (**B**) *Mcap* genome structures were assessed by multiplex PCR and (**C**) pulsed-field gel electrophoresis (PFGE). The annealing sites for the set of ten pairs of PCR primers (Supplementary Table 1) are spaced at intervals of about 100 kb around the chromosome. The sizes of the PCR products from each adjacent primer pair differ in increments of 100 bp, such that the intact *Mcap* genome produces a ladder of ten bands. The multiplex PCR pattern is illustrated here with five yeast clones. Yeast clones were analyzed further by PFGE and produced bands matching the expected size of the PspX1-linearized *Mcap*-CAH genome. Clone 2 of W303a-*Mcap*-CAH was used in subsequent steps. Abbreviations: M, DNA size ladders of 100-1,000 bp (Invitrogen) in panel B, or 225–2,200 kb *S. cerevisiae* chromosomal DNA ladder (Bio-Rad) in panel C; T^{+}, DNA template from *Mcap*-wt cells; T_{H}, negative control without DNA.

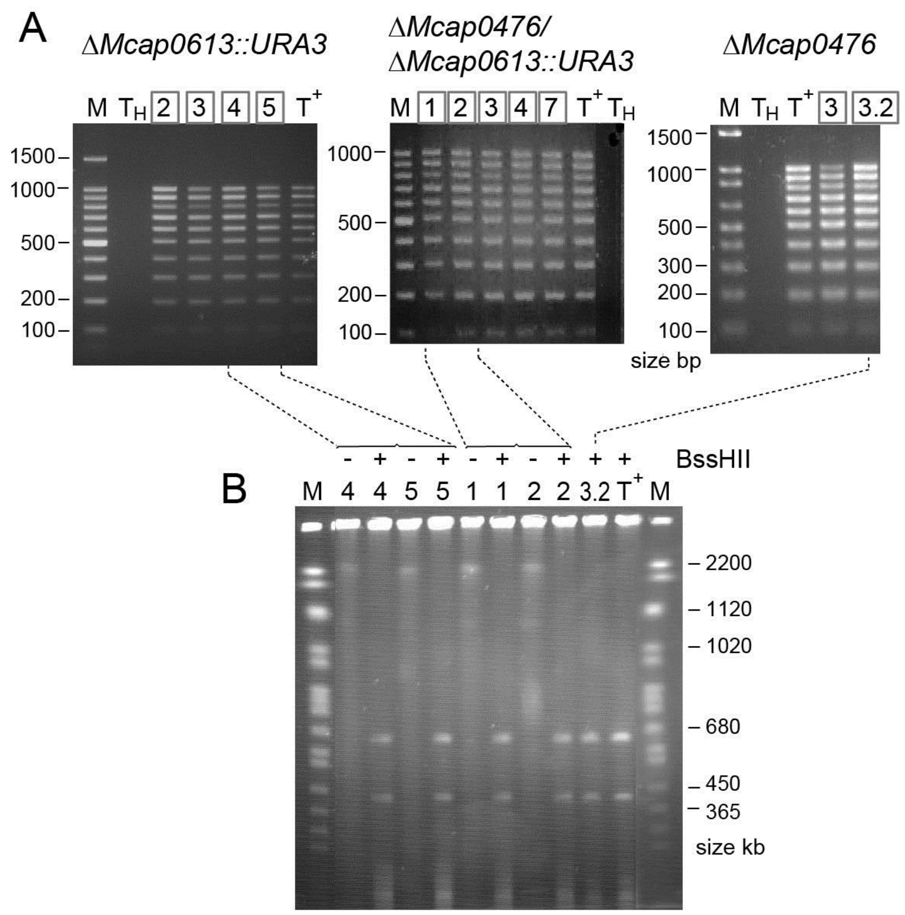

Supplementary Figure 3. Multiplex PCR and PFGE analyses of *Mcap* genomes in yeast. (**A**) Multiplex PCR analyses of yeast clones harboring *Mcap*-CAH genomes with single and double knockouts (Figure 2). The band patterns from these candidates are consistent with correctly structured genomes, and W303-Δ*Mcap0476* (clone 3.2), W303-Δ*Mcap0613*::URA3 (clones 4 and 5) and W303-Δ*Mcap0476/*Δ*Mcap0613*::URA3 (clones 1 and 2) were taken for PFGE analysis (dashed lines). (**B**) PFGE served as one of the final checks of *Mcap*-CAH genome structure and size. After removal of yeast genomic DNA, agarose plugs were either mocked digested (-) or digested with BssHII (+) and separated by PFGE. The *Mcap*-CAH genome contains three BssHII sites (Supplementary Figure 2), two of which are too close together to be distinguished by PFGE; digestion of the selected clones with BssHII gave rise to the expected bands of 389 kb and 621 kb. Bands in the mock digestion are the linear form of the *Mcap*-CAH genome caused by mechanical breaks, and a ~2.2 Mb band from residual yeast DNA. Abbreviations as in Supplementary Figure 2.

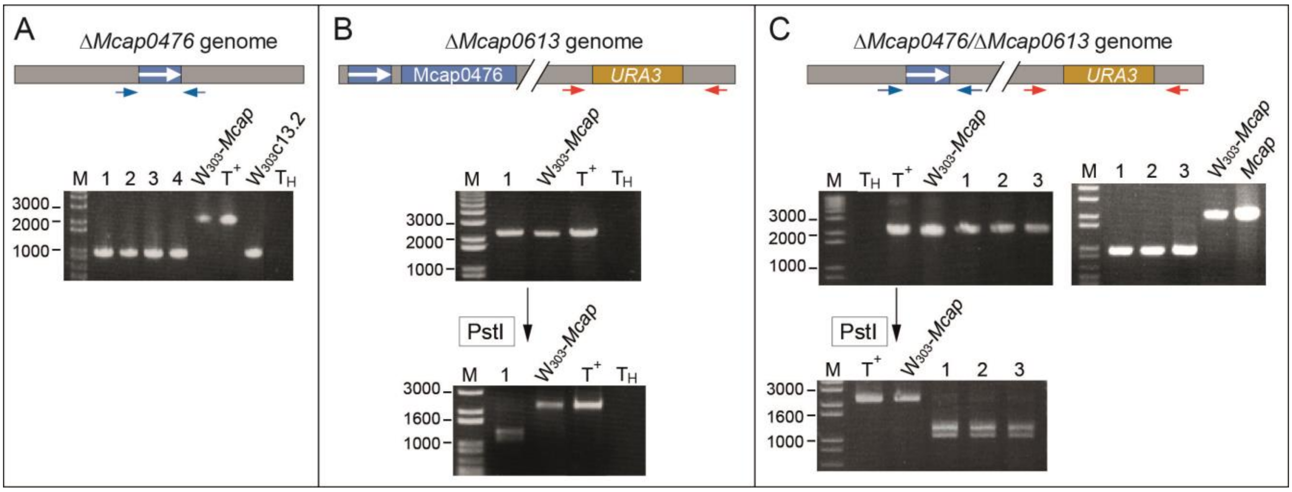

Supplementary Figure 4. Analyses of *Mcap*-CAH genomes after transplantation back into *Mcap* cells. Intact *Mcap*-CAH mutant genomes were isolated from the yeast recombinants W303-Δ*Mcap0476* clone 3.2, W303-Δ*Mcap0613::URA3* clone 4 and W303-Δ*Mcap0476*-Δ*Mcap0613*::URA3 clone 2. The genomic DNA was transplanted back into *Mcap*-wt recipient cells as described above. Deletion of Mcap0476 (Figure 2A) and/or the replacement of Mcap0613 by the URA3 cassette (Figure 2B) was then verified by PCR as shown here for the single knock-out mutants (**A**) *Mcap*Δ*0476* and (**B**) *Mcap*-Δ*0613* and (**C**) the double mutant *Mcap*Δ*0476*/Δ*Mcap0613*. Deletion of Mcap0476 was tested for in panels A and C using the PCR primer pair TrmFO15a and TrmFO16 (Supplementary Table 1) positioned on each side of Mcap0476 (blue arrows). PCR products of 2 kb indicated that Mcap0476 was intact in W303-*Mcap-CAH*, whereas the 1 kb band from clones 1, 2, 3 and 4 showed that Mcap0476 had been seamlessly deleted. Replacement of Mcap0613 by *URA3* in panels B and C was tested with the PCR primer pair TrmFO25b and TrmFO26 (red arrows). Both the *URA3* and Mcap0613 genes produce fragments of 2.3 kb, but can be differentiated by PstI cleavage of *URA3* into bands of 1.0 and 1.3 kb. These mutants were further analyzed by multiplex PCR and PFGE (not shown). Abbreviations; W303-*Mcap*, DNA purified from W303 yeast strain containing a *Mcap*-CAH genome; W303-cl3.2, DNA purified from yeast strain W303-Δ*Mcap0476* (clone 3.2); other abbreviations as in Supplementary Figure 2.

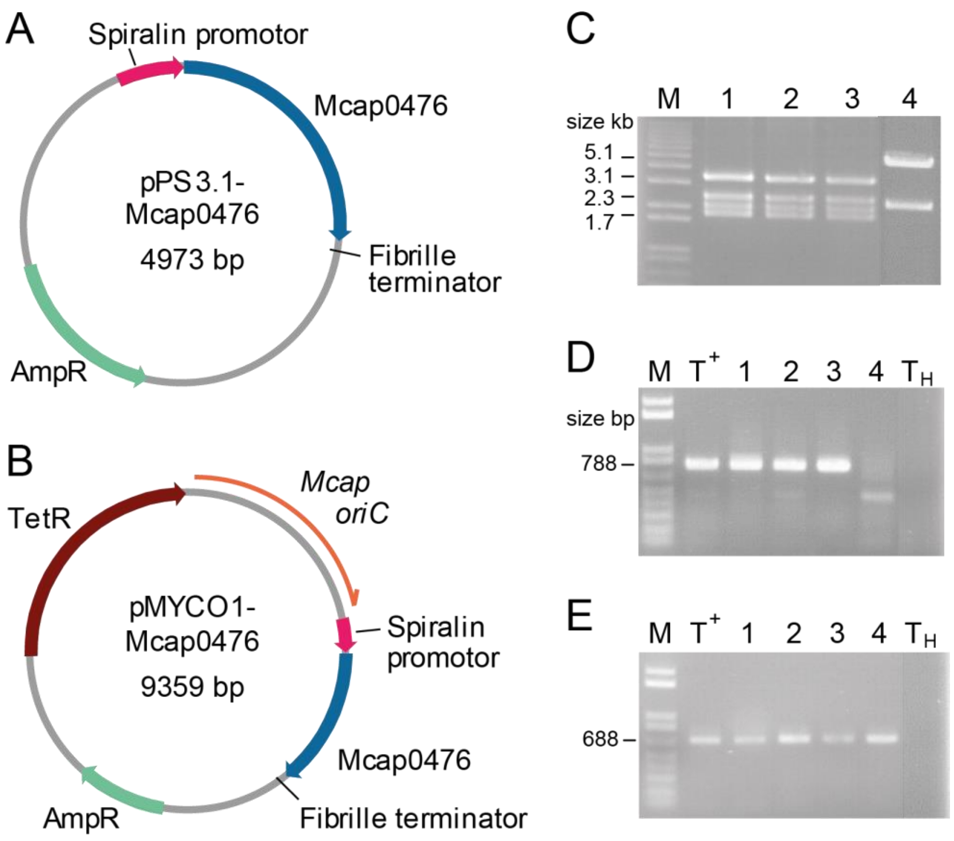

Supplementary Figure 5. Complementation of the *ΔMcap0476* mutant by expression of Mcap0476 from a plasmid. (**A**) Cloning of the Mcap0476 gene into plasmids pPS3.1 and (**B**) pMYCO1 is described in Materials and Methods. Δ*Mcap0476* cells were transformed with the pMYCO1-Mcap0476 plasmid to express the wild-type Mcap0476 enzyme in this knock-out strain. (**C**) After transformation, plasmids were isolated from tetracycline resistant colonies and their structures were tested by PstI restriction analysis. Positive clones produce digestion products of 3.1, 2.3, 2 and 1.7 kb (lanes 1, 2, 3) whereas the empty pMYCO1 plasmid produces two bands at 5.1 and 2.3 kb (lane 4). (**D**) The clones were analyzed by RT-PCR (Materials and Methods) to screen for Mcap0476 expression. Clones 1, 2 and 3 expressed Mcap0476 mRNA giving rise to a cDNA product 788 bp. (**E**) All the four clones produced the Mcap0613-specific cDNA of 688 bp, showing that Mcap0613 mRNA was still been transcribed. Clone 3 was selected for further analysis by MS. AmpR, ampicillin resistance gene; TetR, tetracycline resistant gene; *oriC*, origin of replication; other abbreviations as in Supplementary Figure 2.

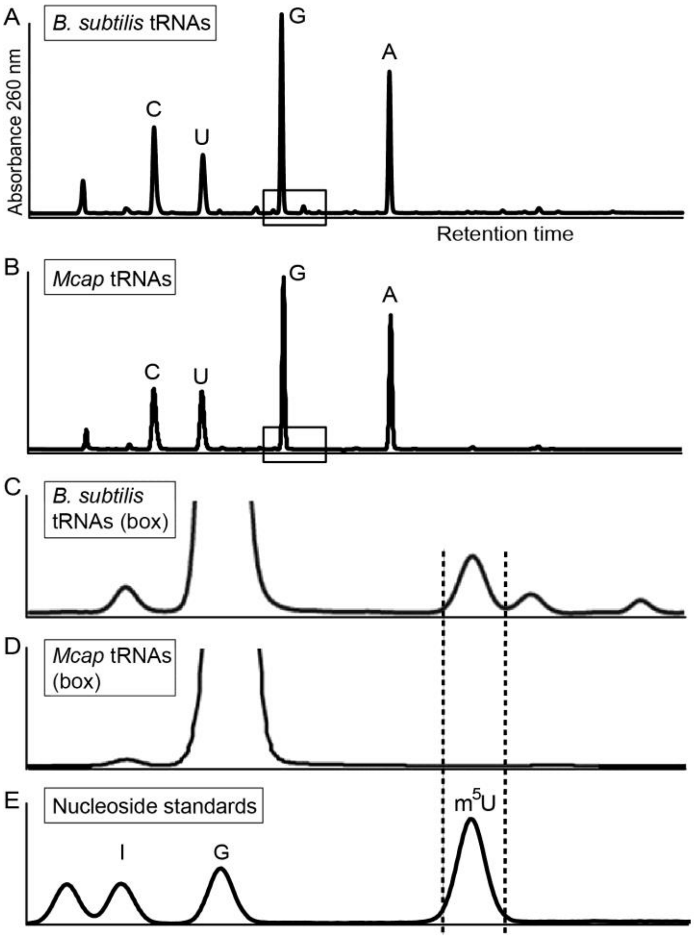

Supplementary Figure 6. HPLC profiles of tRNA nucleosides from bulk *Mcap* tRNA. (**A**) The digestion products of total tRNAs from the wild-type *B. subtilis* strain 168 (7) showing the positions of the unmodified nucleosides. (**B**) HPLC analysis of nucleosides derived from *Mcap*-CAH tRNAs. (**C**) Enlargement of the boxed region containing the *B. subtilis* m^5U modification. (**D**) Enlargements of the same region for the *Mcap* tRNA nucleosides showing absence of m^5U consistent with earlier reports for this species (8,9). (**E**) Nucleoside standardization mixture; the fractions corresponding to the retention times for inosine (I), guanosine and m^5U are indicated.

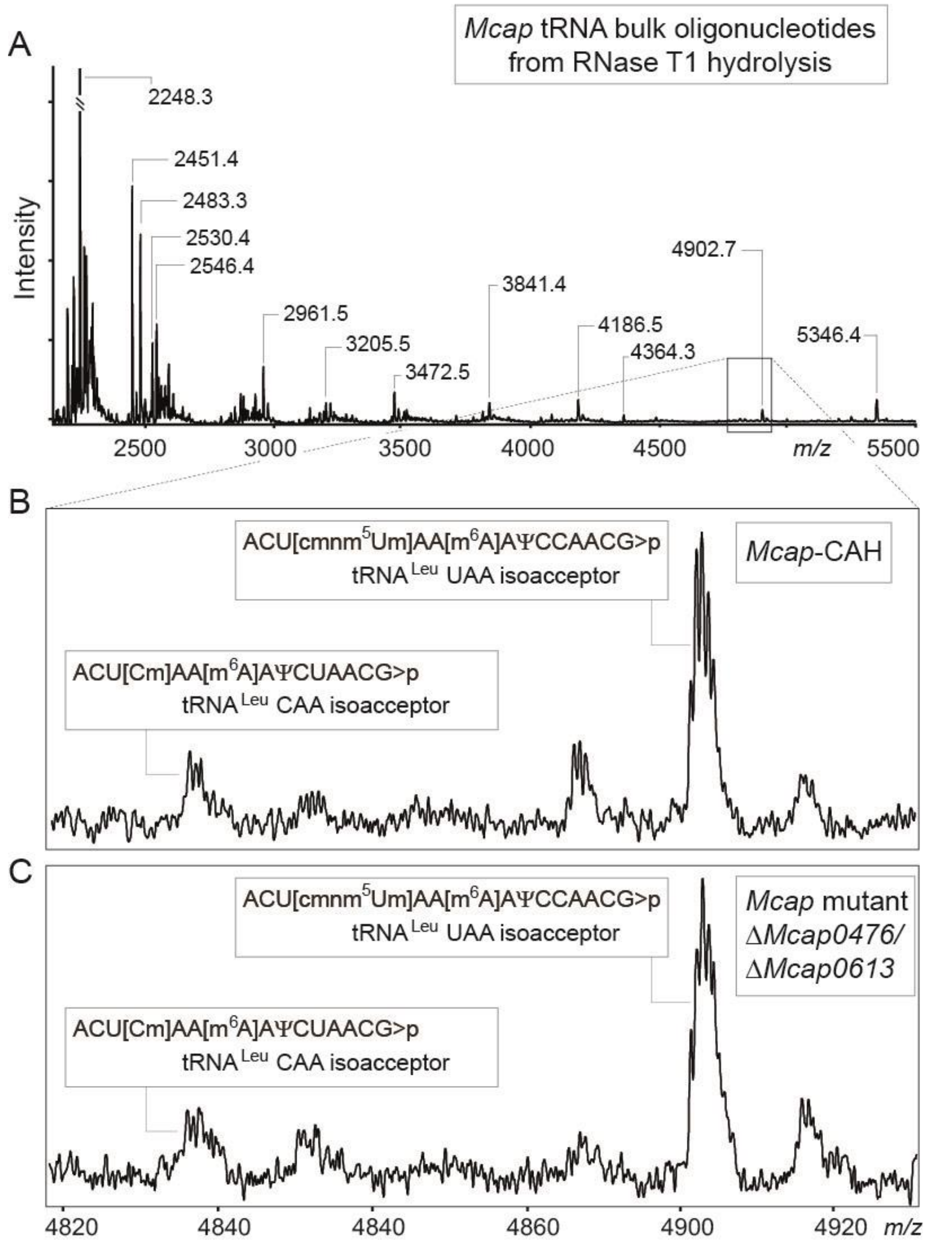

Supplementary Figure 7. (**A**) MALDI-MS analyses of bulk *Mcap* tRNAs after RNase T1 digestion. Oligonucleotides in the range *m/z* 2300 to 5500 are shown, corresponding to heptamers and larger. The $cmnm^5U34$ modification in the $tRNA^{Leu}$ UAA isoacceptor migrates at *m/z* 4902.7 in a 15-nucleotide fragment ACU[$cmnm^5Um$]AA[m^6A]AΨCCAACGp (boxed). This fully modified fragment is clearly evident in (**B**) the wild-type spectrum and also in (**C**) the spectrum from *ΔMcap0476/ΔMcap0613*, as well as from the individual *ΔMcap0476* and *ΔMcap0613* knock-out strains (not shown). The corresponding fragment from the $tRNA^{Leu}$ CAA isoacceptor anticodon at *m/z* 4833.7 lacks this modification, as do all tRNAs with C34 (10). The UCU[$cmnm^5Um$]CA[m^6A]AACCGp (*m/z* 3933.5) fragment from $tRNA^{Trp}$ contains $cmnm^5U34$ in

the wild-type and all the knock-out strains (not shown). All *m/z* values given are for monoisotopic, singly-protonated oligonucleotides with a 2´-3´-cyclic phosphate (11). Most, but not all, of the large RNase T1 fragments could be assigned to predicted *Mcap* tRNA fragment masses including modifications (12). However, some peaks could not be assigned and a few expected peaks were missing indicating that the published list of modifications for *Mcap* tRNAs (12,13) is not fully comprehensive.

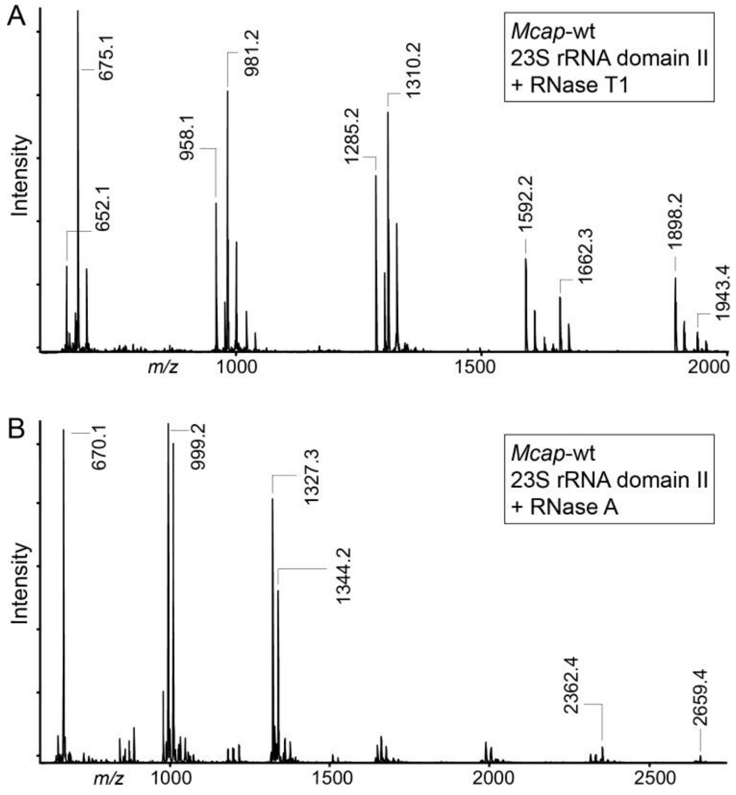

Supplementary Figure 8. MALDI-MS analyses of *Mcap* 23S rRNAs domain II showing the lack of modification at nucleotide U747. (**A**) Spectrum after RNase T1 digestion. Nucleotides U747 and G748 are in the trimer UUG>p at *m/z* 958, and both are unmodified. The *m/z* 958 fragment also contains U746, and the presence of a Ψ746 synthase ortholog in *Mcap* (Figure 3) indicates that this uridine is probably isomerized. Nucleotide G745 is in the fragment UAUUCG>p at *m/z* 1898 and is unmodified. (**B**) Spectrum after RNase A digestion where unmodified G748 is in the fragment GAAAAGACp at *m/z* 2659.

Supplementary Table 1. Additional PCR primer pairs used in this study

Primers for multiplex PCR of *Mcap* genome			
**Primer name**	**Sequence (5´→ 3´)**	**Location in *Mcap* genome**	**Size product (bp)**
Mcap Af<br>Mcap Ar	ATGAACCTAAACGATATTTTAAAAGAACTC<br>TATTAATTGTTTTAATATAGTCGTTATACACAG	1<br>100	100
Mcap Bf<br>Mcap Br	CTAACAGATCCAGCACCAGCTAC<br>TCTTGATATTTTTGCAAAGTAATTTGTACTT	100 001<br>100 200	200
Mcap Cf<br>Mcap Cr	AATTATTGGAATTCAACCAGTAAGTGATG<br>AATTTAGTTGTATTTAAATTAACTCCTTCTTT	200 021<br>200 320	300
Mcap Df<br>Mcap Dr	TTTGATTGTTTCAAATTAACTTTTTAATTGCC<br>GAAATTGGAAAAATTTCTAAAACAATTTCTG	300 011<br>300 410	400
Mcap Ef<br>Mcap Er	CGGCTTGAAAGGCTATTAATGCATCAAC<br>TTGCTGTAGTTACTCATAATCAAAATGTTG	399 931<br>400 440	510
Mcap Ff<br>Mcap Fr	AAGAAGTATAACTCACATACATAGGCG<br>ACATATTTAACATTAATTGTAAATTTTGATTATCTTC	500 101<br>500 700	600
Mcap Gf<br>Mcap Gr	TATTGGTAGATAAGCATGATCAGTACC<br>AATTAGGAAGAGAACCTAATGCTGAAG	600 061<br>600 760	700
Mcap Hf<br>Mcap Hr	AGCTCCAGAAATTACTACAGTTGTTAAG<br>TCCATATTATATTATTGCTCCGCAAATAGC	703 451<br>704 250	800
Mcap If<br>Mcap Ir	CTAAATCTAAAATTCCACCCTCACTAATAG<br>TGCAAGAACTGTTAAAGGTCCAAGAAAAAC	800 061<br>800 960	900
Mcap Jf<br>Mcap Jr	TTAAAGGATTATTTGTTGCTGGTGATGTTA<br>AAATCAACCTAATAATAAGGCAATTGATTCATAG	900 201<br>901 200	1000
**PCR primers for investigating *Mcap* genes**			
TrmFO15a<br>TrmFO16	GAAGTTGGTGGTTTAACTCCAAG<br>CTGAAATTACTCAAGCTTCACC	5´-Mcap0476<br>3´-Mcap0476	1 kb, 2 kb or 3.3 kb
TrmFO25b<br>TrmFO26	GAGTTTGTCAAATTCCATTATCTG<br>CCACTTGCATTGCCATCATTG	5´-Mcap0613<br>3´-Mcap0613	2.3 kb

References

1. Nishimasu, H., Ishitani, R., Yamashita, K., Iwashita, C., Hirata, A., Hori, H. and Nureki, O. (2009) Atomic structure of a folate/FAD-dependent tRNA T54 methyltransferase. *Proc Natl Acad Sci USA*, **106**, 8180-8185.

2. Hamdane, D., Argentini, M., Cornu, D., Myllykallio, H., Skouloubris, S., Hui-Bon-Hoa, G. and Golinelli-Pimpaneau, B. (2011) Insights into folate/FAD-dependent tRNA methyltransferase mechanism: role of two highly conserved cysteines in catalysis. *J.Biol. Chem.*, **286**, 36268-36280.

3. Zhang, Y. (2008) I-TASSER server for protein 3D structure prediction. *BMC Bioinformatics*, **9**, 40.

4. Motorin, Y. and Helm, M. (2011) RNA nucleotide methylation. *Wiley Interdis. Rev. RNA*, **2**, 611-631.

5. Kelley, L.A. and Sternberg, M.J. (2009) Protein structure prediction on the Web: a case study using the Phyre server. *Nat Protoc*, **4**, 363-371.

6. Lartigue, C., Vashee, S., Algire, M.A., Chuang, R.Y., Benders, G.A., Ma, L., Noskov, V.N., Denisova, E.A., Gibson, D.G., Assad-Garcia, N. *et al.* (2009) Creating bacterial strains from genomes that have been cloned and engineered in yeast. *Science*, **325**, 1693-1696.

7. Desmolaize, B., Fabret, C., Bregeon, D., Rose, S., Grosjean, H. and Douthwaite, S. (2011) A single methyltransferase YefA (RlmCD) catalyses both m^5U747 and m^5U1939 modifications in *Bacillus subtilis* 23S rRNA. *Nucleic Acids Res*, **39**, 9368-9375.

8. Hsuchen, C.C. and Dubin, D.T. (1980) Methylation patterns of mycoplasma transfer and ribosomal ribonucleic acid. *J.Bacteriol.*, **144**, 991-998.

9. Johnson, L., Hayashi, H. and Söll, D. (1970) Isolation and properties of a transfer ribonucleic acid deficient in ribothymidine. *Biochemistry*, **9**, 2823-2831.

10. Armengod, M.E., Moukadiri, I., Prado, S., Ruiz-Partida, R., Benitez-Paez, A., Villarroya, M., Lomas, R., Garzon, M.J., Martinez-Zamora, A., Meseguer, S. *et al.* (2012) Enzymology of tRNA modification in the bacterial MnmEG pathway. *Biochimie*, **94**, 1510-1520.

11. Douthwaite, S. and Kirpekar, F. (2007) Identifying modifications in RNA by MALDI mass spectrometry. *Meth. Enzymol.*, **425**, 3-20.

12. Andachi, Y., Yamao, F., Muto, A. and Osawa, S. (1989) Codon recognition patterns as deduced from sequences of the complete set of transfer RNA species in *Mycoplasma capricolum*. Resemblance to mitochondria. *J.Mol. Biol.*, **209**, 37-54.

13. Machnicka, M.A., Milanowska, K., Osman Oglou, O., Purta, E., Kurkowska, M., Olchowik, A., Januszewski, W., Kalinowski, S., Dunin-Horkawicz, S., Rother, K.M. *et al.* (2013) MODOMICS: a database of RNA modification pathways - 2013 update. *Nucleic Acids Res*, **41**, D262-267.
